# Neurocysticercose révélée par une épilepsie réfractaire: à propos d'une observation

**DOI:** 10.11604/pamj.2015.20.104.5958

**Published:** 2015-02-04

**Authors:** Marcellin Bugeme, Olivier Mukuku

**Affiliations:** 1 Faculté de Médecine, Université de Lubumbashi, RDCongo; 2 Centre Neuropsychiatrique Dr Joseph Guislain/Frères de la Charité, Lubumbashi, RDCongo

**Keywords:** Neurocysticercose, Epilepsie, Parasitose, Lubumbashi, neurocysticercosis, epilepsy, parasitosis, Lubumbashi

## Abstract

Nous rapportons une observation de neurocysticercose parenchymateuse chez un homme de 38 ans, consommant régulièrement la viande de porc, qui a présenté une épilepsie réfractaire. Le diagnostic de NCC était basé sur la présence de lésions kystiques montrant le scolex sur les images du scanner cérébrale, la présence de signes cliniques évocateurs de NCC (épilepsie faite des crises convulsives focales évoluant vers des crises bilatérales), la réponse clinique au traitement à l'albendazole et le fait que notre patient vit dans une zone reconnue endémique à la cysticercose. Après un traitement fait d'albendazole et de prednisolone, l’évolution est marquée par la disparition complète des crises épileptiques.

## Introduction

La neurocysticercose (NCC) est une maladie cosmopolite, endémique dans de nombreux pays en voie de développement. Il constitue un problème majeur de santé publique dans plusieurs pays d′Amérique latine, en Afrique, en Asie et même dans les pays industrialisés [1]. C'est une maladie due à l'ingestion par l'homme d’œufs de *Taenia solium* qui se transforment en larves (ou cysticerques) dans l'estomac qui traversent la paroi gastrique pour gagner par la circulation le système nerveux central. Dans le système nerveux central, la larve peut se localiser au niveau du parenchyme cérébral, des espaces sous-arachnoïdiens, à l'intérieur du système ventriculaire et, plus rarement, au niveau de la moëlle et du rachis [2]. La présentation clinique de la NCC est variable dépendant du nombre, de la taille, de la localisation, du stade évolutif du cysticerque ainsi que de la réponse immunitaire de l'hôte. La NCC est une des causes les plus fréquentes d′épilepsie acquise et les crises épileptiques sont le signe plus fréquent variant de 50 à 94,8% [3, 4]. Différents types de crises convulsives ont été décrites chez des patients atteints de la NCC entre autre des crises généralisées, des crises focales et rarement des myoclonies et une aphasie épileptique acquise [5]. L'intérêt de cette observation réside dans le fait que la NCC est une pathologie rarement diagnostiquée dans la ville de Lubumbashi alors qu'elle est située dans une zone fortement endémique de cysticercose.

## Patient et observation

**Figure 1:**
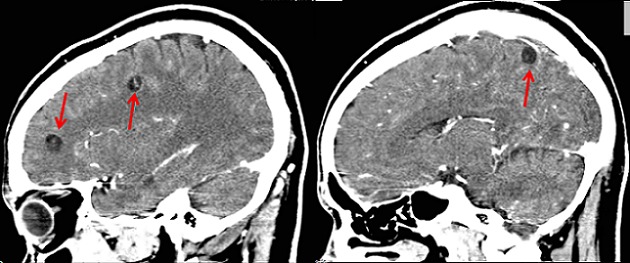
Scanner cérébral montrant multiples cysticerques viables et sans œdème (coupe sagittale)

**Figure 2:**
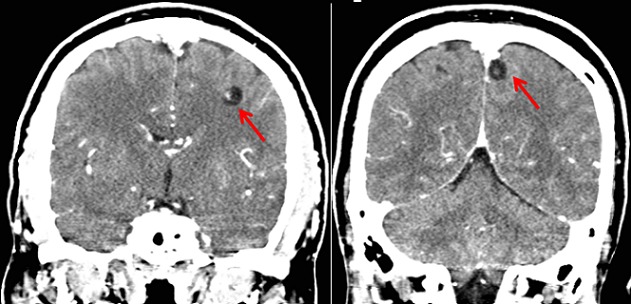
Scanner cérébral montrant multiples cysticerques viables et sans œdème (coupe coronale)

**Figure 3:**
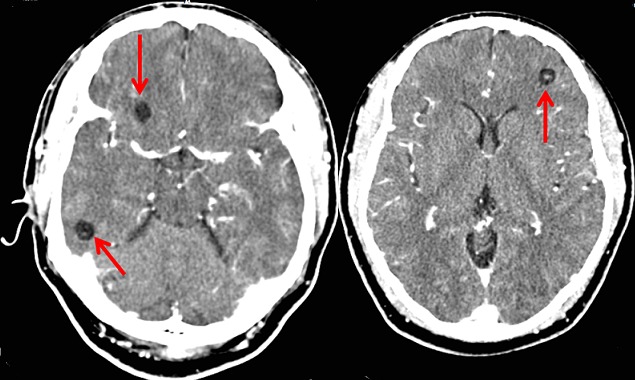
Scanner cérébral montrant multiples cysticerques viables et sans œdème (coupe axiale)

Patient K.M, âgé de 38 ans, présente des crises convulsives qui auraient débuté il y a 3 ans et s'ccompagnaient de chute, de perte de mémoire et d'oubli. Pendant environ deux ans, aucune prise en charge n′avait été instaurée mais une année avant la présente consultation, le patient fut soumis sous un traitement fait de la carbamazépine (deux fois 200 mg par jour). Cinq mois après, vu que la monothérapie ne donnait pas de résultats escomptés, une multithérapie antiépileptique (carbamazépine, phénobarbital et valproate de sodium) lui fut administrée pendant plus de sept mois mais sans succès également. Voyant que son état ne s'améliorait pas, il consulte le Centre Neuro-psychiatrique Dr Joseph-Guislain en date du 21 juin 2014. Ses antécédents ne relèvent rien de particulier et aucune crise épileptique n′a été retrouvée dans sa famille. Mais il faut signaler que dans ses habitus, il consomme régulièrement la viande de porc. Au complément d′anamnèse, nous notons que ses crises convulsives sont focales évoluant vers des crises bilatérales avec des composantes toniques et cloniques. Pendant les crises, il y a une émission d′écume et une perte d'urine. Elles durent environ 5 minutes et ont une fréquence de 2 à 4 par mois. L′examen neurologique était normal. L’électroencéphalogramme montre un tracé perturbé par sa lenteur et par l′abondance en éléments lents à prédominance droite. Le scanner cérébral met en évidence au niveau du parenchyme des multiples lésions kystiques arrondies avec un point hyperdense excentrique représentant le scolex (Figure 1,Figure 2 et Figure 3). Nous avons conclus à une NCC révélée par une épilepsie réfractaire. Après un examen normal du fond d’æil, nous avons instauré un traitement par voie orale fait d'Albendazole (15 mg/kg/jour en 2 prises) et de Prednisolone (1 mg/kg/j) pendant une durée d'une semaine. Un suivi médical pendant une durée de six mois a été fait et aucune crise épileptique n'a été notée jusqu’à ce jour.

## Discussion

Il est difficile de déterminer la prévalence et l'incidence de la NCC en raison de la non-spécificité de sa manifestation clinique et l'absence de tests de laboratoire qui peuvent être utilisés pour confirmer le diagnostic à grande échelle. La majorité des personnes ayant la NCC sont asymptomatiques [6]. Dans une étude menée à Kinshasa (RDCongo), le diagnostic de NCC avait été retenu chez 11 patients sur un total de 4233 scanners cérébraux réalisés en 15 ans soit une fréquence de 0,26% [7]. Malgré que la RDCongo fait partie des pays à haute prévalence de NCC [8], cette pathologie est de diagnostic plutôt rare en milieu urbain. Le diagnostic précis de la NCC repose sur l′évaluation de données objectives de la clinique, de l'imagerie, de l'immunologie et de l’épidémiologie. Ces données ont été regroupées en quatre catégories de critères: absolus, majeurs, mineurs et épidémiologiques [9]. S'agissant de notre cas, le diagnostic de NCC était basé sur la présence de lésions kystiques montrant le scolex sur les images du scanner cérébrale, la présence de signes cliniques évocateurs de NCC (épilepsie faite des crises convulsives focales évoluant vers des crises bilatérales), la réponse clinique au traitement à l'albendazole et le fait que notre patient vit dans une zone reconnue endémique à la cysticercose. Sur le plan histo-pathologique, il y a quatre stades évolutifs de la NCC: vésiculaire, colloïdale, granuleuse (ou nodulaire) et calcifiée [9]. Dans notre cas, l'imagerie est suggestive de l′étape vésiculaire qui apparait comme des petites kystes arrondies qui sont bien délimitées du parenchyme cérébral qui ont dans leur intérieur un nodule hyperdense excentrique représentant le scolex donnant l'aspect d'un “trou-avec-point” (hole-with-dot) tel que le décrit les anglophones, qui est pathognomonique de la NCC [10]. Ce stade vésiculaire est observé lorsque les cysticerques atteignent le système nerveux central et peuvent rester à ce stade pendant plusieurs années [9].

Selon Del Brutto, l’épilepsie et les crises convulsives peuvent se produire à n'importe quel stade histo-pathologique des lésions cysticerques et les mécanismes de l’épileptogenèse varient en fonction du stade. Dans le stade vésiculaire, les crises sont probablement dues aux effets de compression des kystes sur le parenchyme cérébral, tandis qu'aux stades colloïdal et granulaire, les kystes provoquent des convulsions à la suite de la réaction inflammatoire associée à l'attaque du système immunitaire de l'hôte aux parasites. Dans les lésions calcifiées, la gliose qui se développe autour des parasites de la mort, ainsi que l'exposition de matériel antigénique du parenchyme cérébral ou même le développement de la sclérose hippocampique, peuvent être à l'origine de l'activité épileptogène [9]. Le traitement médical est efficace dans les formes parenchymateuses et Matthaiou, dans une méta-analyse, conclut que l'albendazole est plus efficace que le praziquantel dans la maîtrise des crises épileptiques et dans la disparition totale des kystes et, par la suite, dans la guérison des patients atteints de NCC [11]. Son association avec un corticoïde permet de prévenir une réaction paradoxale provoquée par la lyse des parasites en réponse au traitement antiparasitaire qui est responsable d'une réaction inflammatoire à l'origine de l’œdème péri-lésionnel [12].

## Conclusion

Ce cas clinique rapporté met en relief la nécessité de considérer simultanément un certain nombre de critères pour mieux poser le diagnostic d'une NCC. En région d'endémie comme le nôtre, ce diagnostic doit être évoqué de principe devant toute épilepsie de cause inexpliquée et réfractaire à tout traitement antiépileptique.
